# From soil to health: advancing regenerative agriculture for improved food quality and nutrition security

**DOI:** 10.3389/fnut.2025.1638507

**Published:** 2025-10-17

**Authors:** Carl L. Rosier, Anya Knecht, Jasia S. Steinmetz, Amy Weckle, Kelly Bloedorn, Erin Meyer

**Affiliations:** ^1^Basil’s Harvest, Chicago, IL, United States; ^2^University of Illinois Urbana Champaign, Urbana Champaign, IL, United States; ^3^University of Wisconsin-Stevens Point, School of Health Sciences and Wellness, Stevens Point, WI, United States

**Keywords:** regenerative agriculture, ecosystem services, human health, policy initiatives, consumer engagement

## Abstract

Industrial agriculture practices including herbicide-pesticide usage, synthetic fertilizer application, large-scale monocropping, and tillage contribute to increasing concentrations of atmospheric carbon dioxide (CO_2_), exacerbating the effects of global climate change, damaging vital water resources via nutrient pollution and soil erosion, and significantly reducing biodiversity across ecosystems. Observed decadal declines in diet quality driven by industrial farming practices have led to a global health epidemic marked by increased micronutrient deficiency and malnutrition. Additionally, global incorporation of processed foods, a mechanism bolstered by the industrial agricultural complex, contributes to increased prevalence of non-communicable diseases (NCDs), including diabetes and obesity. Regenerative agriculture represents the latest farm management strategy to challenge industrial agricultural methodologies, offering potential approaches to mitigate the myriads of challenges associated with global agricultural food production. However, more than 40 years after redefining a millennium of Indigenous philosophies, numerous barriers continue to limit its large-scale adoption beyond 1% of global farmed acreage. Associated barriers include an unresolved operational definition, lack of standardized certification, and limited research to support both producers and extension specialists. A shortage of systemic collaborative support, including consumer interest and demand, hinders regenerative agriculture adoption. This review examines the global challenges posed by the industrial agriculture model, particularly regarding ecosystem degradation and an inability to meet human nutritional needs. We specifically evaluate the potential of regenerative agriculture to restore global ecosystem services, meet the demands of a growing population, and highlight key knowledge gaps requiring further investigation. Lastly, we identify policy initiatives that, if thoughtfully implemented, could significantly expand the acreage managed under regenerative practices.

## 1 Introduction

Globally, the population is anticipated to peek at 10.3 billion by mid-2080 (1). To meet the projected diet demands of the growing population, agricultural output using current methodologies will need to significantly outpace present production levels (2). However, the World Health Organization (WHO) stated “today’s food systems are simply failing to deliver healthy diets for all” (3). *The State of Food Security and Nutrition in the World 2023* report pinpoints key challenges, including industrial agriculture practices of deforestation, synthetic fertilizer application, tillage, and the large-scale global incorporation of highly processed foods (PFs), coupled with centralized agrifood systems, accelerating both the climate crisis and a rise of non-communicable diseases (NCDs) (4). Regenerative agriculture offers a promising pathway to mitigate greenhouse gas emissions (GHGe) while producing nutrient-dense crops meeting global health and nutrition needs (5). However, significant gaps in quantifiable research and the absence of actionable policy continue to hinder widespread adoption. The central question remains: how can we increase agricultural production sustainably while meeting the nutritional demands of a growing population?

Since the term “regenerative agriculture” emerged in the 1980s, numerous definitions have evolved. Giller et al. succinctly defined it as a set of practices aimed at restoring soil health, capturing soil carbon to mitigate climate change, and reversing biodiversity loss (6). Estimating the global acreage currently managed under regenerative agriculture is difficult due to the absence of a unified certification system, such as the Organic Materials Review Institute used for organic farming. However, if we use organic acreage as a proxy (~2% of global farmland), it is reasonable to conclude that regenerative practices are applied to significantly less than 2% of the world’s agricultural land. Considering that ~37.5% of the Earth’s surface is dedicated to agricultural production, scaling regenerative practices will require expanded research, improved certification, and policy support (7).

Current research offers promising results regarding regenerative agriculture’s potential to enhance ecosystem services by increasing soil carbon storage, enhancing biodiversity, and improving crop nutrient quality, benefits that also support human health. In contrast, industrialized agriculture prioritizes monoculture production, contributing to the global proliferation of ultra-processed foods (UPFs), the concomitant rise in obesity and NCDs, including diabetes and cardiovascular disease, coinciding with the drastic acceleration of ecosystem decline (8, 9). The overreliance on UPFs characterized by high concentrations of added sugars, refined grains, and minimal inclusion of fruits, vegetables, and fish is a direct consequence of the industrial agricultural complex production model. Only nine plants account for 66% of all global crop production (10). This lack of dietary diversity and diminished nutritional quality jeopardizes human health and results in an estimated $10 trillion in associated healthcare costs (11). Diets high in UPFs are associated with micronutrient deficiencies, reduction of gut microbiome diversity, cardiometabolic disease, and mortality outcomes (12–14). Numerous research efforts suggest dietary models that emphasize low-carbon inputs, consisting of greater plant-based foods, and minimal animal protein, reduce GHGe and fulfill human nutritional requirements (15). To reduce dependency on industrial agricultural production of UPFs, action-oriented approaches similar to The Consortium of International Agricultural Research Centres’ (CGIAR) Initiative on Agroecology must be replicated and promoted on a global scale (16).

Numerous policy initiatives such as the Global Soil Partnership, 4 per 1,000, and the Climate Smart Agriculture Alliance have embarked on harnessing the capacity of agricultural soil management as a means of limiting GHGe in order to constrain global temperature increases to 1.5 °C annually in accordance with the 2015 Paris Agreement. Additional policies, including the European Union’s effort to limit pesticide usage to 50% by 2030 and the United States Department of Agriculture (USDA) Conservation Stewardship Program (CSP) and Environmental Quality Incentives Program (EQIP), are assisting in advancing regenerative practices. However, the majority of these policy initiatives do not support small (< 10 acres) to medium (< 1,000 acres) farm operations and are unlikely to achieve lasting change without structural reforms in supply chains, market access, and land tenure systems. Many existing programs are criticized for their prescriptive nature, which does not account for the dynamic and localized challenges farmers face. This rigidity can hinder farmers’ ability to adapt practices in real-time, a necessity given the variability in climate and market conditions. For example, the Midwest Row Crop Collaborative emphasizes the importance of policies that provide flexibility for farmers to choose practices best suited to their operations and local ecosystems while inflexible policies require farmers to face ever-changing conditions necessitating adaptation in real time (17). Policy efforts should incentivize consumer purchasing through improved access, affordability, and education focusing on regeneratively produced foods, thereby driving regional demand and supporting greater local economic growth. For example, Vermont’s farm-to-school programs require the purchase of local, nutrient-dense foods, reinforcing and strengthening regional sustainable foodsheds. Finally, healthcare represents an underutilized avenue for advancing regenerative food systems. Integrating regenerative agriculture principles into medical education and public health strategies can enhance healthcare professionals’ understanding of the link between food quality and chronic disease prevention, shifting healthcare models away from prescription-based solutions.

**Figure 1:**
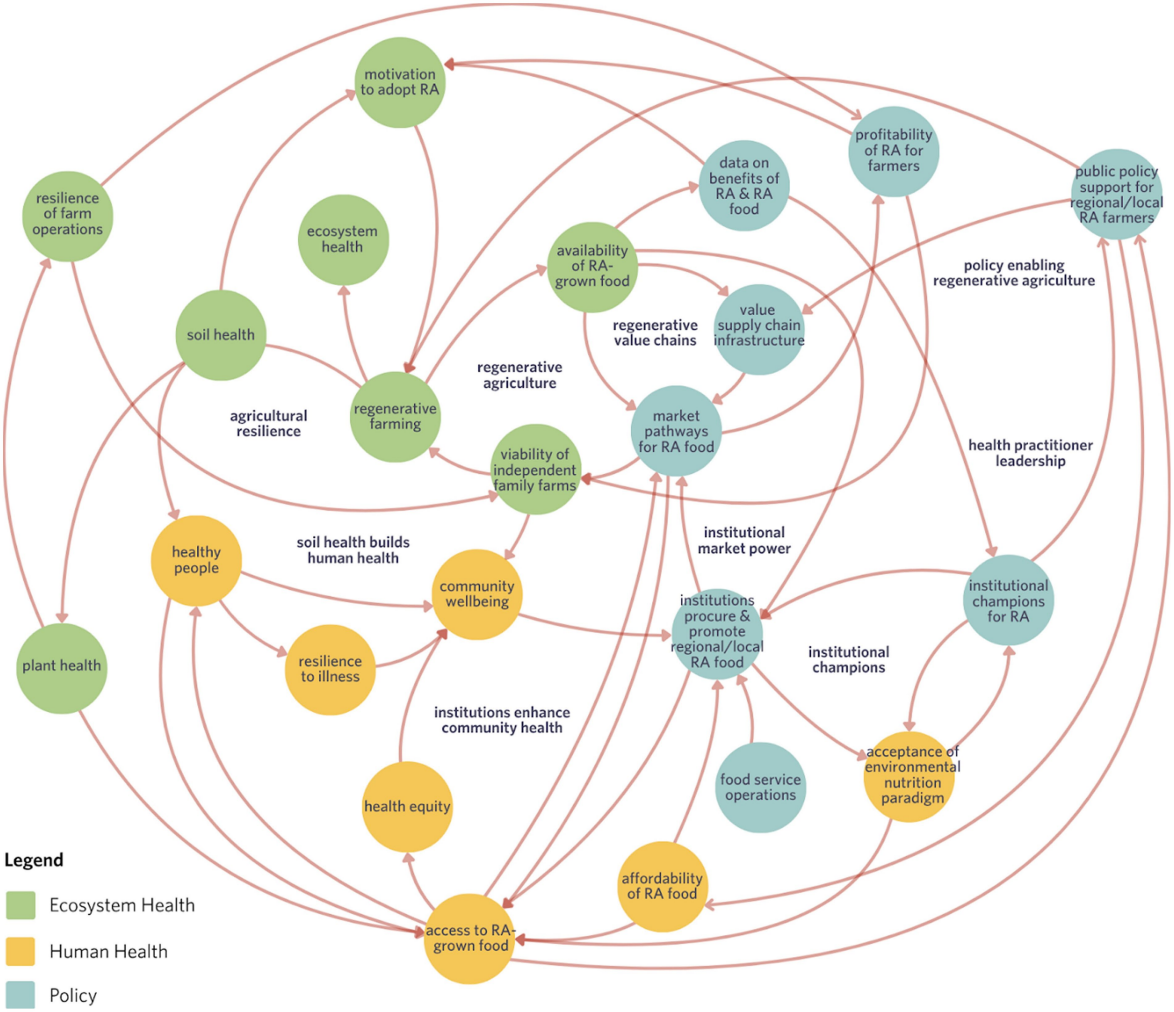
The adoption of regenerative agriculture creates a system of feedback loops restoring both ecosystem and human health and enhancing farm resilience and viability. This leads to a greater supply of nutritious, regeneratively grown crops, opening new markets and supporting independent small-mid range farm operations. Improved plant and soil health benefits farmworkers and surrounding communities, while expanded supply chain infrastructure—including processing and distribution—further supports the growth of regenerative agriculture. Consumer demand and institutional purchasing, especially by hospitals, and schools, drive broader market access and influence policy changes that increase regenerative food affordability and accessibility. Champions within institutions play a vital role in shifting attitudes toward local, regenerative sourcing by promoting the link between farming practices and human health. As regenerative food becomes more available through these channels, it elevates community wellbeing and health equity. Finally, policy support for local regenerative systems assists in counting corporate consolidation within the agrifood sector, protecting the interests of independent producers. Altogether, this interconnected momentum supports a healthier, more resilient, and more equitable food system.

At its core, regenerative agriculture addresses climate change by focusing on soil health and the capacity of the soil to function as a living system (18). Stable, balanced soil ecosystems support nutrient cycling and plant growth while reducing reliance on high carbon-cost inputs that contribute to GHGe and outputs which degrade ecosystems and waterways. Moreover, regenerative practices support human nutrition through the cultivation of nutrient-rich crops, promote social justice by empowering local and regional food systems, and improve food access in underserved communities (Figure 1). Using this framework, this review explores the global challenges associated with industrial agriculture through ecosystem services and food systems lens offering potential policy solutions in order to advance regenerative agriculture practices. Specifically, we aim to:

- Assess the current state of regenerative agriculture, emphasizing the need for definitional clarity and certification oversight, we review the current state of knowledge regarding regenerative agriculture’s potential to increase soil carbon storage, enhance crop nutrition quality and reverse soil biodiversity loss.
- Examine the ability of industrial agriculture, and regenerative agriculture to meet our food and nutrition needs and support human wellbeing during this planetary crisis;
- Assess existing research, policy, and outreach initiatives that would support broader adoption of regenerative practices.

## 2 Methodology

A modified version of the Preferred Reporting Items for Systematic Reviews (PRISMA) method was utilized to identify and organize suitable sources for the development of this review. The PRISMA methodology offers several advantages: (i) systematic identification of relevant literature through well-defined review questions; (ii) transparent application of inclusion and exclusion criteria; and (iii) the ability to evaluate a large body of scientific evidence.

Electronic databases including Scopus, Web of Science (WoS), and Google Scholar were searched for peer-reviewed articles published through April 2025. Search terms were combined with Boolean operators to capture relevant studies with specific terms for each section provided in Supplementary Table 1. Relevant terms were required to appear in the article title, abstract or keywords.

Article selection followed a three-step process: (1) initial screening of article title and abstract; (2) abstract evaluation; and (3) full-text review of selected articles to extract critical results, methodologies and findings of interest.

The search identified 85 possible articles for Section 2: Current State of Regenerative Agriculture, of which 22 were excluded, leaving 63. For Section 3: A Food Systems Approach to Agricultural Evolution, 184 articles were considered and 61 included. For Section 4: Policy Initiatives to Advance Regenerative Agriculture 80 articles were reviewed and 26 met the inclusion criteria. In total, 171 articles were incorporated into this systematic review (Supplementary Figure 1).

## 3 Current state of regenerative agriculture

### 3.1 Definition clarity and unified certification requirements

**Figure 2:**
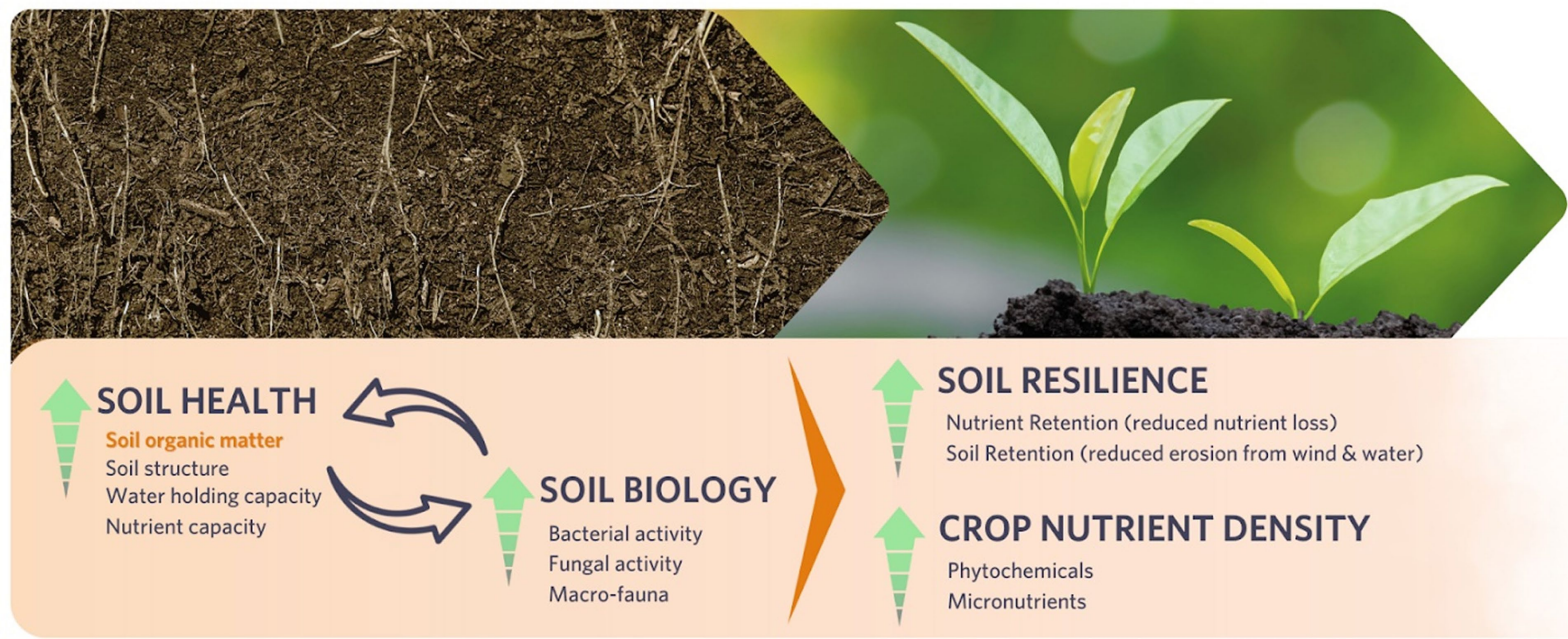
Regenerative agriculture practices including crop biomass retention, cover-cropping, and reduced tillage lead to greater soil organic matter (SOM) accumulation, improving soil structure, water, and nutrient holding capacity. Management practices that increase SOM lead to greater soil food web diversity creating a positive feedback loop where greater enzymatic function within the microbial community assists in continued incorporation of SOM into the soil environment. Research suggests that improvements in the soil environment promote soil resilience to extreme weather events and increase crop nutrient quality.

Alternative approaches to industrial agriculture have been developed and implemented since the mid-century including agroecology, precision agriculture, permaculture, organic farming, conservation, and biodynamic agriculture. Each of these approaches emphasize a specific suite of management practices designed to achieve a particular ecological or agronomic outcome. For example, biodynamic agriculture employs composting, cover-cropping, and natural fertilizers to increase soil fertility, plant health, and animal well-being (19). Among these alternatives, regenerative agriculture has emerged as the most promising, currently positioned at a developmental crossroads and gaining global attention (20). Albeit more than 40 years since the term was reconceptualized by Rodale et al., 1983, techniques associated with regenerative agriculture have been practiced for centuries—if not millennia—by Indigenous communities globally (21). However, there remain several barriers to global adoption. First, the lack of a universally accepted definition that encompasses both general practices and quantifiable performance-based outcomes remains elusive. While some authors have attempted to construct outcome-based definitions that integrate various perspectives across the regenerative spectrum, the continued exclusion of Indigenous knowledge systems reveals a critical gap. This underscores the need for alternative definitions that move beyond Western-centric, value-based frameworks (22, 23). The current consensus suggests regenerative agriculture strives to restore the soil ecosystem services required for sustainable crop production rather than depleting natural resources (6, 22, 24) (Figure 2). However, the lack of a clear definition hinders effective guidance and impedes progress in addressing implementation challenges. Regardless of definition outcomes, further difficulty awaits in selecting, monitoring, and quantifying suitable ecosystem services associated with outcome-based definitions (20, 24). The second barrier to adoption is the absence of a centralized certification or regulatory body, similar to the organizing efforts guiding organic farming—such as the Organic Foods Production Act, the National Organic Standards Board, and the National Organic Program. Currently, two global organizations are leading regenerative certification efforts: the Regenerative Organic Alliance (ROA), and the Savory Institute. The ROA certification process guides agricultural processes and outcomes and is unique in that it allows gradual adoption of regenerative practices providing farmers with an evaluation of bronze, silver, or gold as farm regenerative practices are progressively incorporated into farm management framework and tracking. The ROA also requires standards for social and economic practices. In contrast, the Savory Institute requires short-term and long-term planning for agricultural and ecological outcomes and integrates annual monitoring for achieving goals. Both certification models require monitoring quantifiable indicators such as biodiversity, soil health, and ecosystem function, and provide annual performance reports to farms, a detailed comparison of both certification processes is provided in Supplementary Table 2. The diversity in certification models has both benefits and drawbacks. On one hand, multiple certifying bodies with flexible methodologies can lower barriers to entry by allowing farmers to tailor regenerative practices to regional and operation contexts unlike organic agriculture, which is often criticized for its rigid, uniform standard application across regions that experience different production challenges (25). On the other hand, the lack of a unified definition and regulatory structure exposes the regenerative agriculture movement to “greenwashing” by corporate food and fiber companies, reduces the term to a marketing buzzword lacking substantial meaning, and most critically, undermines the scientific rigor needed to support widespread adoption (26–28). While certification schemes like Regenerative Organic Certified (ROC) and Savory Institute’s Land to Market have emerged to address definitional ambiguity in regenerative agriculture, these frameworks are originally developed within Global North contexts. As such, they may not reflect or adequately incorporate the longstanding regenerative practices embedded in traditional and contemporary agroecological systems elsewhere. Regenerative approaches rooted in Indigenous knowledge have been practiced for centuries in the Global South—promoting soil fertility, biodiversity, and community resilience through methods adapted to local ecological and cultural conditions. For instance, the milpa systems in Mesoamerica, zai pits in the Sahel, and terrace agroforestry in the Andean highlands embody regenerative principles without external chemical inputs (29–31). In more recent decades, movements such as Brazil’s *Movimento dos Trabalhadores Rurais Sem Terra* (MST) agroecology program, India’s Zero Budget Natural Farming (ZBNF), and the CGIAR Agroecology Initiative have demonstrated that regenerative transitions can also be scaled through grassroots leadership and participatory science (16, 32, 33). Recognizing and integrating these systems—both traditional and contemporary—into global definitions and certification efforts is essential for inclusivity, equity, and cross-context learning.

### 3.2 The current state of regenerative agriculture research: soil carbon, crop nutrient density, and soil microbial diversity

Advocates and practitioners of regenerative agriculture assert that widespread adoption could mitigate several global challenges, including reducing climate variability, reversing soil biodiversity loss, and increasing nutrient-dense food production (34, 35). However, the majority of claims lack rigorous scientific validation (27). We aim to address the knowledge gap by evaluating the current state of regenerative agriculture research.

Agricultural soil management practices that enhance carbon capture and sequestration such as biomass retention, cover-cropping, and reduced tillage are considered essential strategies for removing atmospheric concentrations of CO_2_ and mitigating global climate variability (36). In contrast, industrial agricultural systems reliant on petrochemical-derived agrochemicals (e.g., synthetic fertilizers, herbicides, pesticides), biomass removal, and frequent tillage contribute approximately one-third of GHGe, with estimates ranging from 10.8 to 19.1 Gt CO₂eq per year (37, 38). Whereas regenerative agriculture practices focus on soil restoration, increasing soil organic carbon (SOC), and enhancing fertility, and are considered an effective strategy in alignment with global initiatives such as the “4 per 1,000” aimed at the mitigation of GHGe (39). *The World Resources Report: Creating a Sustainable Food Future* suggests that regenerative agriculture offers promise in accumulating SOC (40). A meta-analysis assessing the potential of various regenerative agriculture practices found that diversified crop rotations and managed grazing exhibit the greatest potential for SOC accumulation (0.923–8.388 Mg C ha^−1^ year^−1^), with agroforestry providing 35.178 Mg C ha^−1^ (41). The authors further note that increasing SOC is an important climate adaptation strategy, especially as incidence of extreme weather events (i.e., flooding and drought) are likely to intensify throughout the 21st century. In addition, SOC turnover models using the RothC approach of three regenerative agricultural practices (cover-cropping, reduced tillage intensity, and grass-based ley rotations) across arable land within Great Britain, suggest that 16–27% of GHGe could be mitigated with cover-cropping (9.1 Mg C ha^−1^ year^−1^) and ley-arable rotations (2.7–14.5 9.1 Mg C ha^−1^ year^−1^) (41). However, realistic barriers to widespread adoption remain, including the large-scale implementation of cover cropping and the nitrogen requirements needed to achieve optimal SOC accumulation. The complexity of SOC dynamics—affected by soil texture, climate, and biomass inputs—underscores the need for continued investigation. Notably, only 28 peer-reviewed studies published between 2002 and 2020 have explored regenerative agriculture’s impact on SOC. While the World Resources Institute has proposed federal policy actions to accelerate this research, large-scale implementation has yet to occur (40, 42).

The regenerative agriculture movement has developed a significant following engaging the interest of consumer markets, corporations, and certifiers interested in understanding the connection between crop management practices and nutrient quality (20). Several investigations have noted significant long-term declines in crop nutrient density, potentially due to factors such as the dilution effect, cultivar selection, and industrial management systems (43–45). Significant phytochemical (i.e., antioxidants, phenolic and protein) variation has been observed across leafy greens (lettuce antioxidant content 114-2080 FRAP activity 100 g FW_−1_), root (carrots: antioxidant content 1–67.1 FRAP activity 100 g FW_−1_), and small grain (oat antioxidant: 1,500–3,200 FRAP activity 100 g FW_−1_) crops suggesting that possible mechanisms including cultivar selection, environmental conditions, and management decisions can influence the soil environment (45). In one of the few side-by-side comparative studies, researchers found significant differences in mineral and phytochemical content between industrial and organically managed crops which they attributed to greater soil organic matter (SOM) and increased biological diversity within organic managed soils (35, 46).

It is well established that industrialized agriculture reduces soil microbial community (SMC) diversity and food-web complexity (47). SMC diversity, richness, and functionality provides 80–90% of the soil metabolic activity driving critical ecosystem services such as litter decomposition, soil-C mineralization, and nutrient cycling and are essential to supporting crop production. Diminished microbial genetic diversity has been linked to increases in disease and pathogen resistance within industrial farm soils (39, 48, 49). In addition, increased tillage and application of synthetic fertilizers shift fungal to bacterial biomass ratio favoring bacterial communities, reducing fungal dominance (50–53). Since fungi are essential for the enzymatic processes that incorporate organic matter into soil, their decline limits carbon sequestration capacity (54, 55). Regenerative agriculture practices—such as minimizing tillage, reducing synthetic inputs, and enhancing plant diversity through diverse crop rotations—increases soil microbial biomass and richness, and alters the composition of the SMC structure (56). Increased bacterial diversity has been observed in regenerative vegetable and small grain production plots compared to conventional and barren soil treatments suggesting the rapid recovery of the bacterial community is likely linked to extensive use of organic matter amendments (48). Evaluation of abiotic (i.e., SOM, cation exchange capacity, pH, and water stable aggregates) and biotic (i.e., bacterial and fungal richness, biomass, and functionality) changes in orchard, garden, and pasture soils, sampled from a regenerative working farm located in southern California, across three time points (i.e., 0, 5, and 9 years in regenerative management) indicate an increase in microbial biomass. However, increases in fungal dominance were not detected, possibly due to shifts in saprophytic (increase) and pathogenic (decrease) guilds and no change in bacterial or fungal richness was observed (57). The authors of the study suggest that organic matter amendments were a likely contributor to changes in the SMC, yet, due to the complexity of farm management practices, disentangling an exact set of mechanisms was not attainable. Further support of these observations suggests that consistent soil management practices including reduced tillage and the addition of organic matter amendments promote functional complexity within the SMC (58). When changes in microbial activity, biomass, richness, and community structure were compared between organic and conventional cereal production in a boreal arable soil, differences in crop rotations, tillage, and fertigation practices (organic vs. synthetic) were found to contribute to the observed differences in the SMC (59). Further, the management practices, including the application of manure in the organic treatments, likely impacted temporary differences in soil pH and phosphorus availability. Evidence evaluated to date suggests that interconnected mechanisms of climate, crop variety, management practices, and the degree of SMC degradation prior to regenerative transition likely influence the recovery/shifts in the overall structure and function of the SMC. The use of biofertilizers in combination with microbial inoculants appears to offer promise in accelerating SMC restoration, especially as degraded soils are transitioned to regenerative practices. Further, a greater relative abundance of bacterial and fungal operational taxonomic units have been observed following phosphorus biofertilizer amendments on degraded farm soil (34). The utilization of molecular tools such as next generation sequencing and functional gene analysis offer significant promise in elevating our understanding of the dynamic nature and function of the SMC (57, 60).

Research completed offers promising insight however, additional research supporting observed findings is crucial. Long-term side by side comparative studies that span geographical location, and management practices will be necessary to assess the potential of regenerative agriculture to address outlined global problems. Studies should focus on interdisciplinary research across seemingly disconnected areas of research interest. Our current siloed approach greatly limits the capacity to assess interconnectivity of regenerative agriculture potential to improve global ecosystem and human health. It will be critical to establish investigations that reflect real-world challenges associated with farming while developing requirements of scientific rigor (i.e., replication, comparable treatment models, and accessible analytical methodologies). Funding such initiatives will further offer a unique suite of challenges considering the current political climate and it is likely that non-profit organizations [i.e., 501(c) (3) and non-governmental organizations] will be required to take the lead in advancement of the necessary research.

### 3.3 Factors limiting widespread adoption of regenerative agriculture

Of all human activities, agricultural land management is the most detrimental to the environment, impacting the largest proportion of global ecosystem functions and occupying 40% (~50.4 billion acres) of the earth’s landmass (126 billion acres) (46). Current practices associated with industrial agriculture are detrimental to several ecosystem services. For example, tillage disrupts the capacity of the soil to store carbon, filter water, and cycle nutrients, and application of pesticides has significantly reduced insect populations (i.e., insect apocalypse) (25, 61). A central objective of regenerative agriculture is to first restore and then enhance a range of ecosystem services, most of which are widely recognized as beneficial for sustaining quality of life—though some outcomes may have context-dependent drawbacks (62–65). Investigation into the current deterioration of global ecosystem services and the lack of progress to protect 30% of the planet by 2030 identified economic growth as a key driver of ecosystem service loss and societal values and behaviors as an indirect driver (66, 67). Perception and values assessment of ecosystem services has significant implications governing consumer consumption and production choices that influence degradation (68, 69). Ecosystem service values that are narrowly defined through economic growth tend to dominate, but that perspective ignores non-market values of nature such as those associated with the Indigenous peoples’ and local communities’ worldviews (67). Underpinning the One Health and Planetary Health approaches to achieve sustainable development goals, newly developed valuation processes and methods are needed to equitably evaluate the diverse values of ecosystem services while considering the trade-offs between relevance, robustness, and resource requirements to inform equitable and just strategies and policies (67, 70–72).

Despite the emergence of the organic farming movement in the 1940s, in response to synthetic chemical use and sustained erosional loss of topsoil (i.e., Dust Bowl), and that more than 25 years have elapsed since the founding of the Organic Materials Review Institute (OMRI), agricultural land in organic management only captures an alarming <2% (240 million) of the global acreage. Acreage in transition to or under organic management is increasing, largely driven by Australian rotational grazing operations. However in the United States, organic management acreage has decreased by ~11%, begging the question, “has organic agriculture adoption reached its zenith?” (73, 74). If the regenerative agriculture movement is indeed experiencing a resurgence, what lessons—both successes and shortcomings—from the organic agriculture movement can be leveraged to advance global adoption of regenerative practices beyond the current 2% threshold?

Studies aiming to disentangle both farmer and consumer barriers to regenerative agriculture adoption have found that consumer demand for organically produced crops is the primary driver influencing acreage expansion. However, based on trends in USDA farm census data following evaluation of organic farm sales and land use practices, climatic and economic disruptions, such as droughts and recessions, often result in acreage retractions (74). Surveyed farmers noted significant challenges associated with regulatory oversight, suggesting that overinvolvement by governmental agencies could discourage farmer adoption and long-term participation in organic farming programs (73). Researchers noted that in order to increase organic-regenerative acreage adoption, on-farm research and policy assessments and adjustments will need to address the challenges outlined. Concerns related to the challenges of managing large-scale (>20,000 acres), U.S.-based organic agriculture operations primarily center on issues of profitability, market access, and the limited development of tools and methodologies for effective pest and weed management (75). The study further suggests that a regenerative agriculture certification program should provide flexibility in management options, consider regionality in management decisions, and provide a grower premium, rewarding practices that enhance ecosystem services. One of the few studies evaluating consumer perception developed an organic buyer’s classification system (75). They define two types of organic buyer: committed organic and pragmatic organic. The committed organic buyer is motivated by the philosophy of organic production whereas the pragmatic buyer is driven by personal focus (i.e., price outcomes). Their findings suggest that expanding organic markets will require identifying new committed organic buyers in combination with convincing pragmatic leaning buyers to commit to organic buying. However, a direct mechanistic approach was not presented. Large-scale adoption of regenerative agricultural practices is limited since the majority of industrial farmers are trapped in the agricultural treadmill model of production, experiencing narrow margins coupled with crop price volatility that limit investment in alternative means of production (76). Evaluation of the capacity of organic farming adoption to either break or slow the agricultural treadmill model indicates that greater organic management adoption will only delay the treadmill model (77). The authors note that farmers could enact specific practices improving financial outcomes, such as production of higher quality goods rather than greater quantity, produce commodities that are less price sensitive and restrict overproduction that ultimately leads to lower prices. In order to advance greater adoption of acreage under regenerative management, the connection between food quality and human health will require further advancement including expansion of policy inclusion.

In contrast to the Global North, many smallholder farmers in the Global South face persistent systemic barriers to regenerative transitions—including insecure land tenure, limited access to credit and markets, and insufficient policy support (78, 79). Yet despite these constraints, grassroots agroecological networks have emerged as powerful models of scalable transformation. In Brazil, the MST has embedded agroecology as a foundational principle of land reform and food sovereignty, building community resilience and ecological stewardship across thousands of farming settlements (32). These efforts are increasingly recognized for integrating One Health principles, linking human, animal, and environmental well-being through participatory, localized practice (80). Similarly, in India, the ZBNF movement has successfully mobilized millions of farmers to adopt regenerative, low-cost methods that restore soil health, reduce input dependency, and improve nutrition outcomes—particularly for women and marginalized communities (33, 81). Reviews of agroecological transitions further support these findings, linking traditional knowledge-based systems with measurable improvements in dietary diversity and health outcomes (82). These examples demonstrate how community-led, culturally rooted models can advance regenerative principles at scale—even in the absence of formal certification or subsidy regimes.

## 4 A food systems approach agricultural evolution

### 4.1 Current food system outcomes and human health

Research to address the loss of biodiversity is critical for planetary health and human wellbeing. Although trends in biodiversity loss and ecosystem service declines vary within and among regions, the global human ecological footprint has not halted or been reversed, despite positive efforts to conserve, restore, and sustain biodiversity (83). With an average of approximately 25% of species in assessed animal and plant groups threatened and the rate of extinction rising, understanding and quantifying biodiversity and human wellbeing is gaining recognition (84). A review of regenerative organic agriculture and human health, highlighted the dynamic interaction between soil and plant characteristics that contribute to mutual benefits for ecosystems and human well-being, reduce environmental damage and diet-related diseases while increasing resilience (85).

Conceptual frameworks have modeled interactions between biodiversity and ecosystems, but more recent frameworks include ecosystem services and human wellbeing interactions, leading to a more comprehensive assessment (65, 86, 87). Biodiversity can influence human wellbeing through four pathways: reducing harm (e.g., carbon sequestration, provisioning of food and clean water), restoring capabilities (e.g., stress recovery, attention restoration), building capacities (e.g., physical activity, social interaction and cohesion, transcendent experiences, place attachment and identity), and causing harm (e.g., contact with wildlife or infectious agents, decreased microbiome diversity, exposure to airborne allergens) (86). Local plant knowledge and its value for food security, relationship with nature, medicinal use, ecosystem services, and climate adaptation are shared via sociocultural pathways (88, 89). Seed varieties, application recommendations, and performance metrics of commercial food production are readily available. However, differences in rural, peri-urban and urban pathways for food production and the sociocultural impacts are unknown and informal. Seed sovereignty is a complex local and global issue, given the cultural, social, agricultural and economic interests. Interdisciplinary research approaches that consider sociocultural values and food sovereignty principles would better inform production practices at different scales.

Human health requires agriculture to produce a food supply that supports biodiversity within and between food groups, balances energy, provides adequate nutrient and bioactive compounds important for health across the lifecycle, while maintaining growth, development, and homeostasis without increasing disease risk or compromising earth’s resources for future generations. Our current industrial agricultural production model does not meet these minimal conditions. Healthy diets require a food supply that limits food and beverages that increase the risk of diet-related NCD, including those high in added salt, unhealthy fats, free sugars, non-sugar sweeteners and UPFs (11, 90). Far-reaching, foundational changes are required in order to achieve the global food security and nutrition needs of a growing population that is anticipated to exceed 9.7 billion by mid-century. The changes must consider the dimensions of availability, access, utilization, and stability in the face of increasing climate variability, biodiversity loss, and urbanization and will likely impact numerous aspects of the current food system including production, processing, distribution, and our diets (91). Finally, considering the diversity of environments, sociocultural and economic advantages and challenges, food systems must be responsive and responsible at regional scales. Toward this end, three operational principles to guide regional food systems have been identified by the Higher Level Panel of Experts on Food Security and Nutrition: improve resource efficiency, strengthen resilience, and secure social equity and responsibility (91).

Our current food system approach produces more than the total amount required of dietary calories and nutrients for the global populations, except for choline, calcium and vitamin A, with dietary production and adequacy differing by country (92). Approximately 13.2% of global food produced for human consumption is lost in the supply chain and 19% of food available for retail, food service and consumers was wasted (93, 94). Additionally, global food loss and waste has resulted in a >50% loss of phosphorus, tryptophan, methionine, thiamine and histidine, >40% of the global requirement for 17 out of 29 nutrients were involved in food loss and waste (95). Malnutrition, which includes both undernutrition and overnutrition results in higher disease incidence. Undernutrition is both a determinant and consequence of infectious disease while overnutrition has contributed to increased chronic disease and a global epidemic of overweight and obesity (96, 97). Micronutrient deficiency is the most common condition of undernutrition and can be attributed to inadequate food consumption or declining nutrients levels in foods. An analysis of global food intake across 185 countries observed that greater than half the population does not consume adequate iodine (68%), vitamin E (67%), calcium (66%), iron (65%), riboflavin (55%), folate (54%) and vitamin C (53%) (98). Additionally, concentrations of crop nutrients and beneficial bioactive compounds have been declining over the last 60 years (43, 99). Losses in fruits, vegetables, and food crops include minerals (i.e., sodium, potassium, magnesium, calcium iron, copper, zinc, phosphorus) and vitamins (i.e., vitamins A and C, riboflavin). Potential mechanisms include an increase in mineral nutrient applications, preference for less nutritious cultivars/crops, the use of high yielding varieties, and agronomic challenges related to the shift from organic-sustainable farming to industrial farming operations (64). Dietary micronutrient sufficiency is dependent on an adequate, biodiverse, and secure food supply that is accessible, available, affordable, and nutrient-dense. Lack of access, availability, and affordability of healthy foods coupled with an overabundance of unhealthy foods have put into question our ability to achieve global Zero Hunger by 2030 (4, 11). Estimates of the global quantified hidden costs of agrifood systems exceeded 10 trillion dollars at the end of 2020 largely driven by purchasing power parity with unhealthy dietary patterns, 70% of which are related to costs associated with NCDs (11).

### 4.2 Regenerative agriculture and an ecosystem nexus approach recreates the agrifood system

Arguments against the wide-spread adoption of regenerative agriculture center on the theorized inability to provide food security for a growing global population without significant technological advancement or encroachment into natural ecosystems. However, current industrial agriculture practices that dominate 98% of the global arable land mass will, in all likelihood, experience production declines over the next several decades due to resource scarcity. For example, peak fossil fuels (100) will limit agrochemical production (i.e., herbicides, pesticides and synthetic nitrogen) as well as the operation of the vast assortment of machinery used for planting, harvesting and processing (101). Further, peak phosphorus will reduce crop yields while an estimated 30% of total agricultural land is degraded (102–105). A transitional production model that incorporates organic-sustainable agriculture practices in combination with innovative farming systems has been proposed and may provide the necessary food quality providing global food security; however, it has not been tested to date (106).

**Figure 3:**
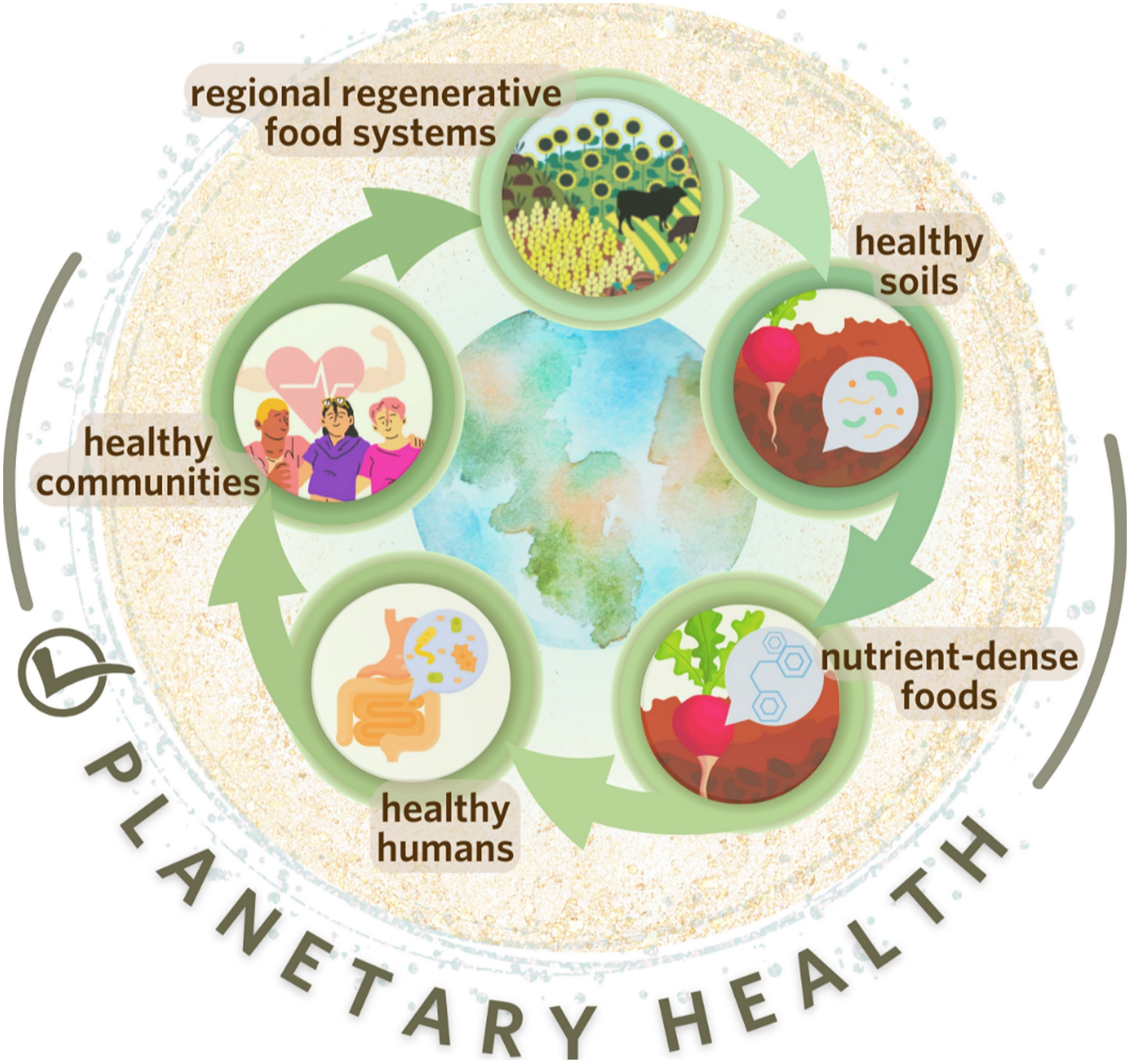
This circular model illustrates how increased adoption of regenerative agriculture can restore and enhance several ecosystem services, increasing both human and planetary health. (i) widespread implementation of regenerative agriculture practices promotes stable, balanced soil environment, increasing the structural and functional diversity of the soil microbiome; (ii) greater nutrient quality of crops prevail with limited to no synthetic chemical interventions, (iii) improved food quality supports greater diversity and functionality of the human gut microbiome, reducing the prevalence of non-communicable diseases; (iv) a healthier population that supports regional regenerative agriculture contributes to local economic resilience and community stability—reflecting a circular economy model.

Regenerative agriculture has demonstrated advantages both for nutrient density of food through increased plant biodiversity as well as increased soil microbial diversity which benefits human microbiome health. The Intergovernmental Science-Policy Platform on Biodiversity and Ecosystem Services has recommended a nexus approach to address the challenges associated with biodiversity loss, water availability and quality, food insecurity, health risks, and climate variability (107). Acknowledging the interlinkages and synergy among specified areas of interest, the nexus methodology addresses both direct and indirect drivers of degradation, holistically, to avoid unintended consequences of isolated efforts supporting transformative change. This suite of guiding principles is closely aligned with Indigenous Peoples and their traditional knowledge, and The Canmore Declaration, a Statement of Principles for Planetary Health (70, 108). A nexus approach challenges the agrifood system to consider its role as integrated connections operating at local-regional levels (Figure 3). This directive evaluates agrifood system requirements for change addressing six of the nine planetary boundaries that have been breached (109). Approaching the nexus with a biodiversity lens connects soil microbiome to human microbiome within the agrifood system and supports the three operational principles for regional food systems.

The ecosystem nexus approach also mirrors the holistic worldview long embodied by Indigenous and peasant farming systems across the Global South. Agroecology movements in Latin America, Asia, and Africa integrate ecological, cultural, and spiritual values that both predate and align with modern regenerative discourse (29, 33). These approaches emphasize not only biodiversity and resilience, but also the importance of honoring knowledge sovereignty and resisting Western-centric paradigms that risk erasing long-standing ecological wisdom. The CGIAR Agroecology Initiative reflects this synthesis by using participatory methods to blend traditional knowledge with scientific frameworks in countries such as Kenya, India, Honduras, and Burkina Faso—demonstrating scalable, regenerative outcomes in both productivity and ecosystem services (16, 110). Furthermore, One Health frameworks adopted in Latin American agroecological movements, such as those linked to Brazil’s MST, illustrate how interconnected health systems—human, animal, and ecological—can emerge through community-led land stewardship (80). Reviews of agroecological interventions globally also show that these transitions contribute to improved dietary diversity and nutrition outcomes, reinforcing the interconnectedness of ecological regeneration and public health (82).

### 4.3 Effect of agricultural practices on the human gut microbiome

The management principles of regenerative agriculture endeavor to establish a diverse and functionally redundant soil microbiome resulting in greater nutrient cycling and reductions in pathogen occurrence (19, 56, 111). Similarly, human wellbeing is significantly influenced by human microbiome diversity and structure particularly in terms of physical and mental health. Current research suggests the microbiome exhibits co-evolution, co-development, co-metabolism, and co-regulation with humans and animals, as well as with humans, animals and bacteria across the evolutionary timeline (112–114). Human microbiome research, especially gut microbiome, suggests biological changes responsive to diet quality and environmental conditions (113–115). Evidence suggesting that environmental biodiversity and human biodiversity are co-developed is supported by the observed transitions of humans from hunter-gatherer to agriculture and domestication of livestock, to urban settings. For example, the microbiome of the Amazonian Yanomami maintains the highest diversity and genetic function ever recorded (116). Specific mechanisms explaining observed results include; remote lifestyle, lack of agriculture or animal domestication and no exposure to antibiotics. As environmental biodiversity declines due to anthropogenic interventions, the co-evolution linkage will inevitably impact human diets, gut microbiome structure, composition and function changes. Case in point: differences in diversity and composition of the human gut microbiome were found in populations experiencing different lifestyles (e.g., hunters-gatherers, pastorals, agropastorals, agriculturists and urban dwelling) (117–119).

**Figure 4:**
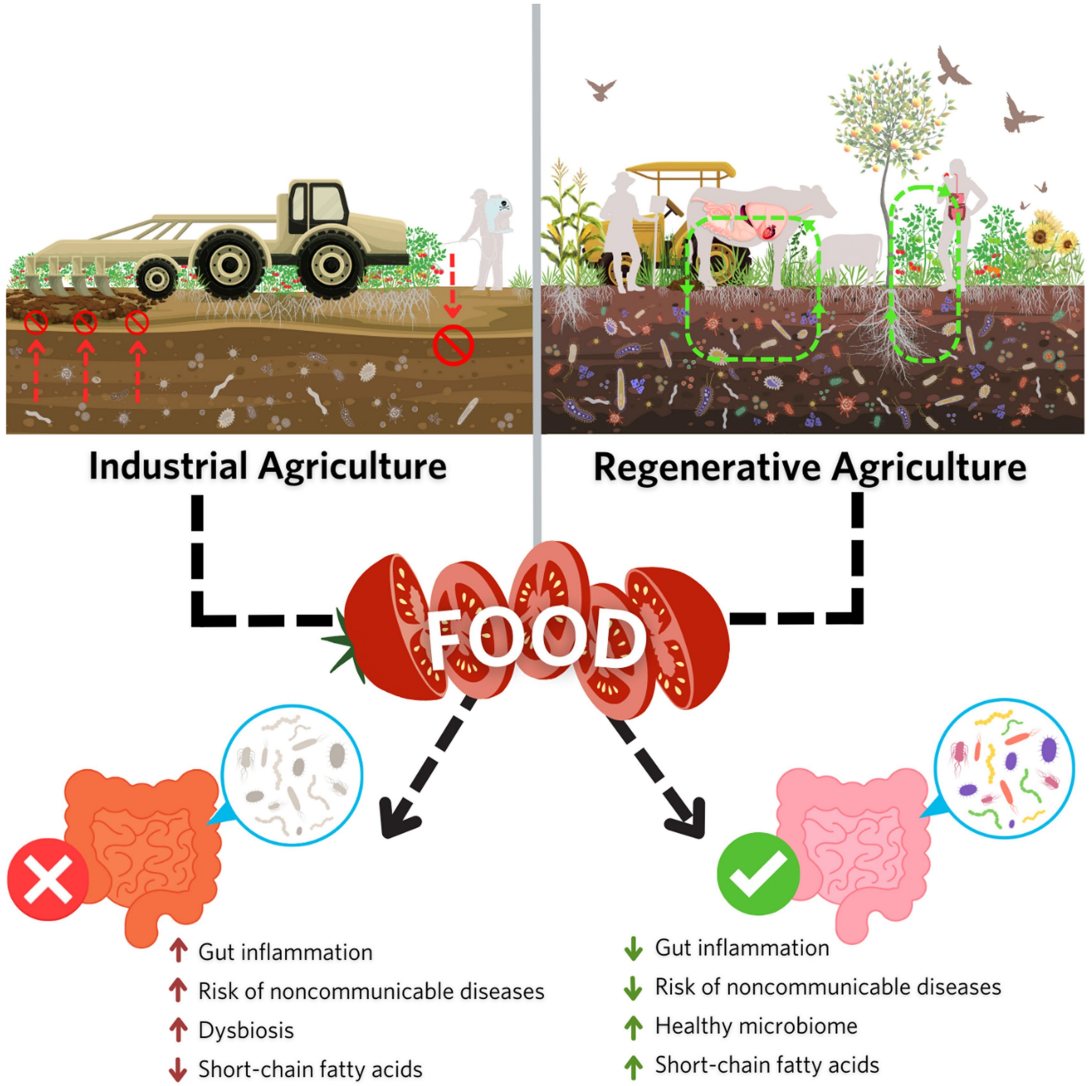
Research suggests that differences in soil quality such as the accumulation of soil organic matter and microbial diversity and functionality influence crop quality including mineral content and phytochemical concentrations, potentially impacting human gut microbiome health. Regenerative agricultural practices prioritize the restoration and maintenance of the soil environment, improving crop nutrient quality, and supporting a more diverse and functional human gut microbiome. In contrast, industrial agricultural practices, which rely extensively on synthetic inputs, have been shown to negatively impact soil microbiome diversity, potentially leading to negative downstream effects on human gut microbiome composition and health.

Current research efforts assessing the influence of food quality and diet composition on the human microbiome, specifically the human gut microbiome, have established preliminary connections between health maintenance and disease prevention (Figure 4). Results from a study of 4,930 participants with elevated health food choice scores, as recommended by the Nordic Nutrition Recommendations dietary guidelines (i.e., omnivorous diet rich in plants, fiber and polyunsaturated fatty acids), suggest that observed participants had greater diversity and compositionally distinct individual gut microbiota (120, 121). These findings suggest protective factors in preventing non-communicable chronic disease and support a plant-based diet. The four-year Shanghai Women’s Health Study (*n* = 74,940) and Shanghai Men’s Health Study (*n* = 61,49) collected dietary selection data samples across 0 (baseline), 2, and 4-year intervals offering important insights into the long-term influences of diet quality and food selection on gut microbiome diversity and function. A follow-up study collected stool samples, histories, and food frequency questionnaires of 144 (4.5%) participants from the original study; generating a Healthy Diet Score (HDS) based on eight food groups: positive scores in fruits, vegetables (excluding potatoes), dairy, fish and seafood, nuts and legumes; negative scores in refined grains, red meat and processed meat (122). Results suggest that higher HDS is associated with increased alpha-diversity of fecal microbial gene families and metabolic pathways related to increases in cofactor, carrier, and vitamin biosynthesis and the tricarboxylic acid cycle. Microbiome analysis results were confirmed with a cohort of 1,600 southwest Chinese Han participants. Findings identified a reduction in overall diversity of bacteria associated with higher Dietary Approaches to Stop Hypertension scores. Overall results suggest a plant-based diet, with attention to fruits, vegetables, whole grains, beans, and nuts is critical to gut microbiome structure and function (123). The multigenerational observational Framingham Heart Study (FHS) in combination with the FHS Generation 3 study, evaluated spouses and second generation participants, who were not related to the original FHS generation, yet reside in the same community (124). Based on recall, the diets of the 1,356 participants included the same foods as the Shanghai study with the addition of yogurt/active bacterial cultures, probiotics, and white meat (122). Dietary factors, including increased overall diversity and consumption of fish, vegetables and fruit, were positively associated with the greatest gut microbiome diversity whereas processed meat and dairy were inversely associated. Additional findings suggest that diets high in meat and dairy were associated with bacterial groups known to be anti-inflammatory through short chain fatty acid (SCFA) production. The increasing evidence of a plant-based diet supporting a healthy gut microbiome and reduced cardiovascular disease suggests regenerative production methods could provide these essential diet components.

An overall assessment of global dietary patterns suggests that rural and urban differences in the gut microbiome are attributed to specific microbial group functionality. For example, rural populations have increased Bacteroidetes, *Prevotella* and *Xylanibacter*, Gram-negative bacteria that degrade polysaccharides and complex carbohydrates (125, 126). Differences in the diversity and resulting functionality of the gut microbiome may also be attributed to seasonal variations in rural areas in contrast to the consistent foods available in urban settings. Microbiome changes have been noted in the Hudza gut microbiome with variation between wet and dry seasons (127). Similar seasonal observations have been made in Hutterite populations of North Dakota as well as Mongolian nomads, middle-aged Japanese, and a cross-sectional study in Ukraine where diets are higher in fiber-rich fruits and vegetables in the summer as compared to the winter season (125).

Despite the evidence that population health requires a diverse plant-based diet, coupled with traditional dietary patterns including a variety of whole or minimally processed foods, especially UPFs are becoming increasingly prevalent throughout food systems. UPFs are ready-to-eat or heat formulations prepared by assembling food substances consisting mostly of heavily subsidized commodity crops (i.e., high fructose corn syrup, soybean oil, rice, and wheat) and ‘cosmetic’ additives (i.e., salt and chemical flavorings) utilizing a series of industrial processes (128). UPFs are designed to be maximally addictive and hyperpalatable in order to disrupt satiety signals and increase consumption (129, 130). It is not surprising that the rise of NCDs in the global population coincides with the increased prevalence of UPFs. Health outcome population studies suggest the changes in the gut microbiome, upon greater UPF consumption, leads to gut dysbiosis which is associated with inflammation and can result in a lower presence of SCFA-producing bacteria and an increased permeability of the gut or a reduced overall bacterial diversity (131). UPFs are a result of increasing industrialized advancement of the agrifood system and a shift to complete reliance on a narrow number of crops. This shift provides an outlet for subsidized commodity crop production including processed meat products, and feedlot animal and poultry production. A Brazilian household food survey found that household food baskets with a higher content of UPFs were associated with a significant reduction in the diversity of available agricultural products (132). Diets that consist primarily of UPFs contribute to food selection homogenization (i.e., convergence of the Western diet). Currently approximately 255 plant species are cultivated for food, globally, with three staple crops providing 60% of the global calories (133, 134).

### 4.4 Research requirements: advancing our understanding of connections between food systems and agricultural practices

Given the increasing global costs associated with NCDs and the proliferation of UPFs, the question remains “can our agrifood system respond given our triple planetary crisis of pollution, biodiversity loss and climate variability?” (135). Most importantly, regenerative agriculture cannot be sustained, given this crisis, or support human wellbeing, given the current consolidated food system, without a system approach. Assessment and action that moves past silos and considers multiple factors across the system is necessary at this watershed moment. Half of the earth’s habitable land is used for agriculture suggesting that food production efforts that address this suite of crises are critical (136). Indicators have been developed for farmland habitat that capture various levels of global biodiversity, but the Agrobiodiversity Index is a more comprehensive assessment across the food system and includes conservation, production, consumption areas management practices, and policy commitments that support food system changes (136, 137). A more robust Index would signal increased availability in the food system, which would require successful efforts to diversify global diets and reduce the dominance of the Western Diet, thereby reducing NCD incidence. Regional and seasonal diet studies have not been conducted longitudinally to correlate gut microbiome changes and significance to health.

Attempts to capture food systems in urban and peri-urban areas as well as accounting for the different pathways and policies affecting the rural–urban continuum have proven more challenging (11, 138, 139). Significant changes to the food system must address true costs, which account for environmental, social, and health factors as a result of market concentration and lack of institution or policy accountability (11, 140). Studies that assess these costs and efforts to address them require increased funding commitments and transdisciplinary teams.

Recognizing and accounting for the hidden costs and benefits of regenerative agriculture requires integration and development of a more robust system monitoring and assessment model. The Farmscape Function framework, the Agrobiodiversity Index, and Tools for Agroecology Performance Evaluation (TAPE) consider agriculture and environment considerations as well as social and economic factors (137, 141, 142). A global monitoring system capable of local adaptation would support SDG assessment while encouraging local innovation and climate responsiveness to guide research at local to national levels.

Interdisciplinary and collaborative research approaches are necessary to address the triple planetary crisis. The CGIAR Agroecology Initiative underway in eight countries takes a transdisciplinary, participatory and action-oriented approach that incorporates science, practice and social movements (16). Engaging more stakeholders within the communities and across systems may mobilize more people for innovations and behavior change, increase support for farmer expertise and stewardship, and increase equity and economic parity.

Although numerous policies and programs exist to support sustainable and regenerative agriculture, their formation and implementation have often occurred in silos, reflecting fragmented priorities across economic, environmental, health, and social domains. To move from isolated success stories to coordinated transformation, we must identify and address systemic gaps in existing frameworks. Supplementary Table 3 summarizes a diverse array of U.S. and global policy efforts and proposals, categorized by impact area, showing the breadth—but also the disconnection—of current initiatives.

The table also reveals four critical barriers that persist despite recent progress:

- Lack of policy support for farms under 1,000 acres, who are often excluded from incentive structures and disproportionately impacted by certification burdens.
- Absence of unified certification standards, with limited consensus beyond Regenerative Organic Certified (ROC) and Land to Market programs, weakening transparency and market trust.
- Minimal financial and structural support for land transition, including a lack of tax incentives, land access programs, and risk mitigation tools for first-time regenerative adopters.
- Absence of integrated health-agriculture policy frameworks and integration into practices, pharmaceuticals, food is medicine prescription programs, despite clear evidence linking food quality and chronic disease prevention.
- Insufficient mechanisms to adjust consumer behavior, such as reward structures, institutional procurement reform, or education-driven incentives that reflect the true value of nutrient-dense, ecologically produced food.

To advance regenerative agriculture meaningfully, these gaps must be addressed through cross-sectoral coordination, equity-centered reform, and integrated policy mechanisms that reflect the ecological, economic, and human health stakes of current agricultural practice.

## 5 Policy initiatives required to advance regenerative agriculture

### 5.1 Expanding policy inclusion across the smaller farm operation spectrum

Globally, policies aimed at promoting sustainable agricultural practices have evolved significantly over the past few decades. The European Union’s (EU’s) Common Agricultural Policy (CAP) has implemented financial incentives and regulatory frameworks to encourage organic farming, agroecology, and environmental-friendly practices. For instance, the EU’s Green Deal and Farm to Fork Strategy aim to reduce chemical pesticide use by 50% through incentives for integrated pest management, agroecological transitions, and reduced-input farming systems. Further, through the use of subsidy reallocation, labeling support, and expanded research and advisory services, this policy aims to increase organic farming to 25% of agricultural land by 2030 (143). Internationally, initiatives such as the FAO’s Global Soil Partnership, which promotes sustainable soil management through coordinated global action and national policy frameworks, and the 4 per 1,000 Initiative, which advocates for increasing global soil organic carbon stocks by 0.4% annually as a climate mitigation and food security strategy, have set benchmarks for soil carbon sequestration and soil health improvements (11). In the United States, programs such as the USDA’s Conservation Stewardship Program (CSP) and Environmental Quality Incentives Program (EQIP) have provided financial support to farmers transitioning to regenerative practices (144, 145). However, the majority of federal agricultural policies to date have prioritized large-scale monoculture commodity farming—a model that has clearly been linked to long-term soil degradation, biodiversity loss, and rural economic decline. While programs like CSP and EQIP represent progress, their reach remains limited by administrative complexity, eligibility barriers, and misalignment with the needs of small (<10 acres) and medium scale (<1,000 acres) diversified farms. As summarized in Supplementary Table 3, a range of global, national, and state-level policy mechanisms have been developed to promote regenerative practices, though most remain underutilized by small and mid-sized farms. Furthermore, many regenerative benchmarks and soil health goals risk becoming aspirational rather than actionable if they rely solely on short-term financial incentives without systemic reform. Under the current industrial agricultural framework, these subsidies may become unsustainable or unenforceable once funding lapses, unless they are accompanied by structural shifts in supply chains, market access, and land tenure and transition systems that embed regenerative practices into the fabric of everyday farming. Additional challenges persist, including the need for clearer certification standards and enhanced outreach to small (< 10 acres) and medium-scale farmers (< 1,000 acres). Specifically, the term “regenerative” lacks universally recognized certification criteria, leading to inconsistencies in labeling and market trust. Efforts like the Regenerative Organic Certified (ROC) standard and Savory Institute’s Land to Market program represent emerging frameworks that attempt to codify regenerative practices across soil health, animal welfare, and social fairness dimensions, but broader consensus and institutional recognition are still needed to ensure credibility and equitable access. Increasing regenerative agriculture adoption will require systemic shifts in production incentives, and consumer involvement in collaboration with nuanced policy initiatives.

Altering our current agricultural management trajectory will involve developing support mechanisms for diverse, regional farming operations that prioritize soil health, water conservation, and biodiversity. Beyond perceived gains in ecosystem services, additional increases in both economic prosperity and improved human health outcomes of local communities could come to fruition with the departure from conventional agricultural practices. For example, communities located near large-scale industrial farms often experience economic extraction, where profits are funneled out of rural areas rather than reinvested. Furthermore, proximity to conventional operations is associated with increased rates of respiratory illness, toxic pesticides, antibiotic resistance, and other health burdens due to air and water pollution from concentrated animal feeding operations (CAFOs), pesticide drift, and nutrient runoff (146, 147). A regenerative approach offers an alternative path, reversing these trends by rebuilding ecological and physiological resilience from the ground up. By fostering improved soil health and increasing microbial diversity, regenerative systems have been shown to enhance the nutrient density of crops which, when incorporated into diets, can mitigate NCDs such as diabetes, cardiovascular disease, and hypertension. Ultimately, adoption of regenerative practices will reduce public health expenditures, improve human health, and the quality of life (148, 149).

### 5.2 Policy advocacy increasing consumer engagement

Increasing regenerative agriculture practices will require change in behaviors and conscious decision-making at both the policy and consumer level. Examples of programs that advance initiatives to increase regenerative agriculture adoption are numerous. Recently, California’s Healthy Soils Program demonstrates the potential of state-level proposals to incentivize climate-smart agricultural practices by providing direct grants to farmers and ranchers for implementing soil-building practices (cover-cropping, compost application, reduced tillage, hedgerow planting) alongside technical assistance and quantifiable metrics to track greenhouse gas reductions and soil carbon sequestration (150). Expanding similar models nationally could enhance the scalability of regenerative agriculture while supporting small landowner and immigrant farming communities, as observed with the United Farm Workers Union’s advocacy for improved labor conditions (151). This effort brought attention to the intersection of environmental stewardship and farmworker rights, leading to increased visibility, policy alignment, and funding for programs that prioritize both soil health and equitable working conditions. Additionally, consumer demand plays a pivotal role in driving regenerative agriculture adoption. Policies that enhance access, affordability, and education regarding whole or minimally processed regenerative products can significantly impact market dynamics. For example, farm-to-school programs that mandate local, nutrient-dense food procurement have proven successful in states such as Vermont, increasing children’s access to healthier meals while supporting local agricultural economies (152). Globally, public food procurement initiatives that promote sustainable development, such as those in school feeding and school meal programs, public hospitals, prisons, universities and cafeterias and other social programs, and consider social, economic and environmental values have resulted in increased agrobiodiversity and encouraged agroecological production; empower rural producers, including smallholder and women farmers; increased resilience and nutrient-density of food networks; and reduced carbon footprints (153–157). However, consumer barriers persist, particularly regarding price perception and food preparation knowledge areas, requiring further evaluation.

While structural change in policy is essential, shifting behaviors—particularly at the consumer and institutional levels—remains one of the most significant barriers to widespread adoption of regenerative agriculture. Behavioral inertia is reinforced by decades of exposure to ultra-processed, convenience-driven food systems, limited food literacy, and marketing practices that normalize cheap, high-yield, low-nutrient products. Many consumers face real or perceived barriers to preparing whole or minimally processed foods, including time constraints, lack of culturally relevant options, or inadequate infrastructure such as grocery store access or kitchen tools. When available, regenerative products may be overlooked if consumers are unfamiliar with how to use them, and they may not associate them with added health or ecological value. Overcoming these behavioral barriers will require more than information campaigns; it will involve investing in community food education, cooking literacy, institutional trust-building, and economic incentives that make regenerative food choices not just available—but easy, desirable, and aligned with daily routines. Programs that pair incentives with experiential learning—such as community-supported agriculture shares bundled with meal kits, or school gardens linked to cafeteria meals, hospital meals or prescription medication from regeneratively grown produce—can help bridge the gap between knowledge and sustained behavior change. Leveraging resources that are currently in our system as a vehicle to make these choices feasible can be a mechanism to changing our daily behaviors. Creating programs that help with land transition and provide tax breaks for farms can support these changes. Supplementary Table 3 summarizes many existing and proposed regenerative agriculture policy initiatives across different scales and domains, highlighting their impacts, policy types, and implementation levels.

### 5.3 Policy initiatives must collaborate across stakeholder groups

To support widespread adoption of regenerative agriculture, policy frameworks must center the voices and experiences of farmers. This includes developing adaptable, incentive-based policies that prioritize soil health, biodiversity, and long-term sustainability—designed with direct input from farmers themselves to ensure feasibility and relevance across diverse operations (158). At the same time, healthcare systems have an essential role to play. Integrating regenerative agriculture principles into medical education and public health strategies can strengthen the connection between food quality and chronic disease prevention, shifting healthcare models toward more holistic, nutrition-driven approaches (149). Finally, regenerating our food systems will require a reimagining of community infrastructure. Urban–rural partnerships must be cultivated to foster resilient local/regional food systems through community-supported agriculture, land access initiatives, and regionally—tailored food system planning that empowers both producers and consumers (139). Many of the recommended reforms—such as procurement targets, cross-sector incentives, and healthcare integration—are reflected in Supplementary Table 3, which maps current initiatives and gaps across policy types.

Historical and current policies reveal the potential and pitfalls of promoting sustainable agriculture (as noted above).

- Increasing regenerative agriculture production requires a shift from monoculture-centric incentives toward soil health-based policies.
- Consumer demand hinges on education, accessibility, and the economic viability of regenerative products.

#### 5.3.1 Future research needs

- Quantitative studies measuring regenerative practices’ long-term impacts on soil health and human nutrition (106).
- Sociocultural analyses exploring consumer behavior changes and adoption patterns (158).

#### 5.3.2 Policy recommendations

- Enhance certification clarity for regenerative agriculture practices (11).
- Increase incentives for regeneratively-grown food in markets and institutions.
- Expand state-level programs [ex: California’s Healthy Soils Program nationwide (150)].
- Integrate regenerative agriculture topics into public health, education, and policy discourse (149).
- Mandate that 30% of produce comes from a locally—sourced farm for schools, hospitals, groceries, and restaurants. Models, for example, Good Food Purchasing Program in Los Angeles and New York City have already set procurement benchmarks of up to 25% for sustainably or locally sourced foods, demonstrating the feasibility and public benefit of institutional leverage (159). Similarly, New York’s Farm-to-Institution initiative has implemented a 30% NYS Food Procurement Incentive for schools, reinforcing the value of public resources reshaping food system priorities. Kenya, Guatemala and India offer local diverse foods in their school feeding programs and Brazil will pay a premium of up to 30 percent in the price of organic or agroecological produce (160).
- Offer an incentive program for conventional industrial farmers to transition >5 acres of land to being regenerated and utilized for a local educational space (4H, FFA, etc.) to change the landscape for food production according to the region’s soil profile, processing geographically, and given markets (school, hospital, grocery).

Collaborative efforts across sectors must be developed in order to achieve a resilient, nutrient-dense, and environmentally sustainable agrifood system. By assessing global policy successes and addressing persistent challenges, stakeholders can facilitate a transformative shift from conventional agriculture’s extractive practices to a regenerative, health-centered model. However, systematic and widespread change in agriculture practices cannot rely on top-down policy alone, it must include shifts in consumer behavior that incentivize decisions and reinforce change. Examples include choosing regionally sourced or regeneratively grown products, supporting community-supported agriculture (CSA) programs, participating in local food co-ops, hubs, and aggregators, or advocating for institutional procurement policies that prioritize regenerative and regional sourcing in schools, hospitals, military, and government programs. These behaviors act as market signals that incentivize producers and retailers to shift supply chains accordingly. Beyond food purchasing, consumer demand can also reshape expectations in healthcare. As more individuals seek dietary prescriptions and nutrition-based interventions to prevent and manage chronic illnesses, this pressure will put the impetus on physicians to incorporate food-based solutions into their clinical workups alongside traditional pharmaceutical regimens. This growing movement toward food-as-medicine may also prompt pharmaceutical companies to explore the therapeutic potential of nutrient-dense, regeneratively grown foods. If a fraction of the resources currently allocated to health insurance premiums and prescription drugs were redirected into our communities, supporting local/regional food systems and access to regenerative nutrition—we could simultaneously rebuild economic resilience and human health. In 2023, the CDC estimated that NCDs—including heart disease, diabetes, and obesity—account for $4.1 trillion in annual U.S. healthcare costs, much of which is preventable through dietary and lifestyle interventions (161). While aspirational, redirecting even a small portion of these expenditures toward regeneratively—grown, nutrient-dense food systems could yield transformative public health outcomes and economic relief. Such reinvestment holds the potential to restore gut microbiomes, enhance cognitive and emotional wellbeing, strengthen immunity and cellular repair, and deepen our collective capacity to recover from illness and stress. In essence, this presents the opportunity to regenerate, survive, and sustain.

### 5.4 Shifting the paradigm to advance regenerative agriculture adoption

Realizing this vision will require a deeper understanding of how everyday choices connect to long-term consequences, and the removal of structural barriers that limit choices. This will require more than individual awareness—it will demand a structural rethinking of how food choices are shaped, constrained, and enabled by broader systems. Consumers cannot be expected to drive change without equitable access to information, time, resources, and affordable, value-aligned alternatives. In many communities, especially those historically marginalized or economically underserved, regenerative options are simply not present—or recognizable—due to lack of infrastructure, outreach, and culturally relevant education. These limitations are not due to individual failures, but rather systemic design: our current food system fosters a one-track, industrialized model that rewards efficiency, profit, and yield over quality and equity, and it funnels both economic and health outcomes into extractive cycles.

To shift this paradigm, we must invest in what behavioral economists call “choice architecture.” Simply stated, we must redesign environments so that regenerative and health-supporting options are the easiest, most accessible, and most desirable paths forward and are rewarded through repetition of choice (162, 163). This includes institutional procurement policies that normalize regenerative products in public settings (i.e., schools, hospitals, and government programs), food labeling that emphasizes nutrient density and soil impact, and healthcare platforms that connect dietary guidance directly to regenerative supply chains (155, 164). Longevity requires that individuals are not only empowered to make better decisions, but that the systems implemented are designed to support and sustain those choices.

Advancing regenerative agriculture must not replicate the inequities embedded in our current food production/distribution system. Food justice and equity demand that all communities have the knowledge, access, and agency to make choices aligned with their health, environmental, and cultural values (165). This requires investments in equitable food infrastructure, such as urban gardens, food hubs, mobile markets, and culturally relevant nutrition education, especially in communities historically marginalized by industrial agriculture (166, 167). Institutions can play a powerful role in this transition by incorporating regenerative procurement standards into public programs including Supplemental Nutrition Assistance Program (SNAP), school meals, and healthcare-based food interventions (168). For example, farm-to-school programs have demonstrated success in simultaneously improving nutrition for children and supporting local producers, while advancing racial and economic equity (152, 169). Additionally, case studies such as the Food Security Partners coalition in Tennessee show how participatory, cross-sector coalitions can successfully advance community food security by building local capacity and reshaping relational structures within the food system (167). For example, urban gardens have been shown to increase food access, social cohesion, and even property values while simultaneously enhancing biodiversity and mitigating climate impacts (170). By illuminating the environmental and health consequences of food selection, and by ensuring that affordable, attainable, and dignified alternatives exist and are readily available to all, we can encourage consumers to choose regional, regeneratively grown foods.

Our current food production system fosters one-track, monocropping, and industrialized solutions that restrict dietary autonomy and discourage participation. Currently we reward many of the behaviors that perpetuate a cycle of dependency on unsustainable systems that diminish both soil ecosystem function and human health. Rebuilding a resilient agrifood system requires reimaging consumer choice as an act of agency, and providing the resources, infrastructure, and institutional support needed to make that agency actionable and inclusive.

## 6 Conclusion

Regenerative agriculture offers a compelling framework for restoring ecosystem health, mitigating the effects of climate change, and producing nutrient-rich food for a rapidly growing global population. However, unlocking its full potential requires more than isolated practice—it demands systemic transformation. A clear, inclusive definition that reflects the diversity of agricultural contexts, coupled with a flexible yet unified certification framework, is essential. Expanded research must guide farmers and extension agents, while educational and behavioral shifts—driven by policy and consumer engagement—are necessary to transition from awareness to action. Achieving meaningful, lasting change will also depend on addressing inequities in access and reforming supply chains to support sustainable practices. Ultimately, multi-stakeholder collaboration across farmers, policymakers, healthcare professionals, and consumers is critical to creating an enabling environment in which regenerative agriculture can move from the margins to the mainstream.
