# Supplementary material for: From soil to health: advancing regenerative agriculture for improved food quality and nutrition security
Source: Front Nutr. 2025 Oct 17;12:1638507. doi: 10.3389/fnut.2025.1638507 (PMC12576041; doi:10.3389/fnut.2025.1638507)
Supplement: Supplementary file 1 [file Table_1.docx]

**Supplementary Table 1:** **Search string used for the systematic review process by section**

| **Section** | **Keywords** |
| --- | --- |
| 3.1 | “Regenerative agriculture” AND “Definition” AND “Certification OR“ “Indigenous philosophy” AND “Limitations” |
| 3.2 | “Regenerative agriculture” AND “Soil Health” AND “Carbon Storage OR “Sequestration” OR “Soil Organic Carbon” OR Greenhouse Gas Emissions” AND “Nutrient Cycling” AND Soil Microbial Community Structure” OR Diversity” OR “Redundancy” AND “Crop Nutrient Quality” |
| 3.3 | “Regenerative agriculture” AND “Acreage application” OR “adoption” AND “Ecosystems Services” AND “Farmer Engagement “AND Consumer participation” “One Health” AND “Ecosystem” OR “Indigenous” OR “Local” |
| 4.1  4.2  4.3  4.4  5.1  5.2  5.3  5.4 | “Food Security” AND “Nutrition” AND “Agriculture”, “Human health” AND “Biodiversity” OR “Plant biodiversity”, “Human Wellness” AND “Biodiversity”, “Biodiversity” AND “Ecosystem” AND “Human health”, “Food system” AND “Food Security” AND “Nutrition”, “Food security” AND “climate change” AND “Diet quality”, “Agroecology” OR “organic agriculture” AND “Food Security” AND “Nutrition”; “Sustainable Development Goal” AND ”Food systems” OR “Nutrition”  “agriculture” OR “regenerative agriculture” or “organic agriculture” AND “resource scarcity” AND “food security”, “agroecology” AND “planetary health” AND “agri-food systems”, “Indigenous knowledge” OR “traditional knowledge” AND “planetary health”, “planetary boundaries”.  “Microbiome” AND “Diet” AND “agriculture”, “soil microbiome” AND “human microbiome”, “food quality” AND “microbiome” AND “Diversity”, “Ultraprocessed foods” AND “microbiome  “agrobiodiversity assessment”, “SDG assessment” AND “biodiversity”, “agroecology assessment”, “regenerative agriculture” AND “assessment”  “Regenerative agriculture” AND “Policy” OR “Legislation” OR “Regulation” OR “Certification” OR “Standards” AND “Adoption” OR “Implementation” OR “Assessment”  “Regenerative agriculture” AND “Incentives” OR “Subsidies” OR “Financial support” OR “Tax policy” OR “Land access” OR “Transition support” OR “Resource scarcity”  “Regenerative agriculture” AND “Consumer demand” OR “Market development” OR “Institutional procurement” OR “Food systems policy” OR “Supply chains” OR “Equity”  “Regenerative agriculture” AND “Healthcare” OR “Food as medicine” OR “Nutrition policy” OR “Public health” OR “Medical education” OR “Chronic disease prevention” “Human wellness” AND “Biodiversity” OR “Ecosystem” AND “Human health” |

**Supplementary Table 2: Comparison of Regenerative Organic Alliance and Savory Institute Ecological Health Regenerative Certification Practices**

|  | **Regenerative Organic Alliance** (1) | **Savory Institute Ecological Health** (2) |
| --- | --- | --- |
| **General Conditions** | ***Input and outcome based*** | ***Index (outcomes based)*** |
| **Eligibility:** | USDA organic certification or international equivalent recognized by the U.S. National Organic Program required | May vary by ecoregion |
| **Non-Eligible practices** | Soilless practices: aquaponics, hydroponics and other soilless. Minor exceptions for some water plants | None specifically mentioned |
| **Regenerative requirements** | Minimum of 3 required. Examples include: agroforestry, silvopasture, mulching, perennial planting | Minimum requirement not specifically addressed |
| **Evaluation methods** | **Goal**: increase SOC and sequester carbon, improve animal welfare, provide economic stability and fairness for farmers, ranchers and workers.  **Measurements (**bronze, silver, or gold):   - soil health lab test and soil field tests; - computer models to determine GHG emissions and sequestrations; - records of native flora and fauna on farm as Key Performance Indicators; - record of soil lab test and in-field soil recommended | **Goal:** varies by ecoregion and addresses ground cover, water infiltration, biodiversity, primary productivity, soil carbon & health.  **Current application**: land bases with livestock operations, producing meat, dairy, wool and leather.  **Measurements** (short- and long-term evaluations required):   - vegetable and water filtration change: bare soil cover, litter cover, foliar cover of perennial plants by species, cover percentage by functional groups, biodiversity indicators such as Species Richness or Shannon Wiener Index, water infiltration; - soil carbon and health evaluated; - short-term monitoring generates Ecosystem Health Score |
| **Soil management** | **Goal**: minimal soil disturbance, increase SOC and carbon sequestration **Recommendation(s):**   - Tillage action plan; - Increase litter abundance, incorporation, and decomposition - Organic certification practices: compost, animal and green manures, cover crops, mulches, conservation tillage, contour plowing and strip cropping, crop rotation, no synthetic pesticides or herbicides are allowed, buffer zones from conventional agriculture to prevent contamination. | **Goal:** minimal soil disturbance  **Recommendation(s):**   - Reductions in tillage - Avoidance of bare soil events and soil capping - Limit wind and water erosion - Increase litter abundance, incorporation, and decomposition |
| **Manure management** | **Recommendation(s):**   - Establish facilities for the management of wastewater, and manure - Land application of compost & manure as part of fertilizer practice plan | **Recommendation(s):**   - Land application of compost and manure as part of fertilizer practice plan. |
| **Water conservation** | **Recommendation(s):**   - Restore natural bodies of water, wetland, riparian areas and associated habitat; - Legal irrigation rights. - See soil management | ***See***: *soil management* |
| **Forest/Tree management** | **Goal:** deforestation operations have not cleared forested areas (primary untouched, old-growth secondary; converted wetlands, peatlands, protected grasslands) | **Goal:** Vigor and reproduction of a functional group  **Recommendation(s):**   - Encourage trees and shrub growth - Live canopy abundance |
| **Management of Flora and Fauna** | **Goal:** Control of invasive species; protection of endangered plants and animals  Recommendations:   - Organic seeds and planting stocks - No GMO or treated with prohibited substances allowed. - No drift from the prohibited pesticides or fertilizers | **Goal:** Vigor and reproduction of a functional group  **Recommendation(s):**   - Warm season grasses Cool season grasses Forbs and legumes Reduce presence of contextually undesirable species - Encourage development/stability of micro & macrofauna |
| **Grazing practices** | Rotational grazing recommended. Continuous confinement is not allowed. Must eat certified organic feed. | Rotational grazing required |
| **Worker and Animal Standards** | Farmer & Worker Fairness standards required. Animal Welfare standards required | Farm Worker fairness standards not specifically addressed.  Animal Welfare standards required |
| **Additional notes** | Use of prohibited substances: organically approved pesticides are controlled or others not allowed.  Extractive practices: no fracking, mining or other extractive practices |  |

**Sources**

1. Becoming Regenerative Organic Certified® [Internet]. Regenerative Organic Certified. [cited 2025 Aug 7]. Available from: https://regenorganic.org/becoming-regenerative-organic-certified/

2. EOV Summary [Internet]. [cited 2025 Aug 11]. Available from: https://savory-institute.gitbook.io/eov-manual-public/~gitbook/pdf?page=e4Fs2jRLMO5X427ik6IZ&only=yes&limit=100

3. United State Department of Agriculture. Introduction to Organic Practices. {Internet} Available from: https://www.ams.usda.gov/publications/content/fact-sheet-introduction-organic-practices

**Supplementary Table 3: Overview of policy initiatives advancing local/regional sustainable-regenerative adoption**

| **Policy/Program Name** | **Years** | **States/ Regions** | **Type of Policy** | | | | | | | **Impact(s)** | **References** |
| --- | --- | --- | --- | --- | --- | --- | --- | --- | --- | --- | --- |
|  |  |  | Economic | Agricultural | Environmental | Social Advocacy | Procedural/ Regulatory/ Educational | Health/ Nutrition | Food System/ Procurement |  |  |
| EU Common Agricultural Policy (CAP) + Farm to Fork Strategy | 2020–2030 | European Union | x |  | x |  | x |  |  | Incentivized organic farming, targeted 25% land in organic by 2030, 50% pesticide reduction | (1) |
| FAO Global Soil Partnership & 4 per 1000 Initiative | 2012–Present | Global |  | x | x |  | x |  |  | Benchmarks for soil carbon sequestration; national policy frameworks for soil health | (2) |
| USDA CSP & EQIP | 2002–Present | USA | x | x |  |  |  |  |  | Support for regenerative practice adoption, limited by administrative hurdles and misalignment with small/medium farms | (3,4) |
| Regenerative Organic Certified (ROC) & Land to Market | 2018–Present | USA/Global |  | x | x | x | x |  |  | Emerging frameworks for soil health, animal welfare, social fairness; not yet universally accepted; Trying to obtain some consensus on RA metrics | (5) |
| California Healthy Soils Program | 2017–Present | California, USA | x | x | x |  |  |  |  | Grants for soil-building practices, GHG reduction, supports small and immigrant farmers | (6) |
| Farm to School - Vermont | 2010s–Present | Vermont, USA | x | x |  |  |  | x | x | Increased local procurement in schools, healthier meals, local farm support | (7) |
| Good Food Purchasing Program (Los Angeles, NYC) | 2012–Present | Los Angeles, New York City, USA |  | x | x |  | x |  | x | Procurement standards targeting 25% sustainable/local sourcing | (8) |
| NYS 30% Procurement Incentive | 2020s | New York State, USA | x | x |  |  |  |  | x | Increased local food procurement for schools | (9) |
| United Farm Workers Advocacy | 1960s–Present | USA |  | x | x | x |  | x |  | Improved labor conditions; increased visibility and alignment of labor and environmental policies | (10) |
| CDC Chronic Disease Cost Analysis | 2023 | USA | x |  |  |  |  | x |  | $4.1 trillion in annual costs from chronic diseases, supporting need for food-as-medicine policies | (11) |
| Food as Medicine Initiatives (Healthcare Integration) | 2020s–Present | USA |  | x |  |  |  | x |  | Promotes regenerative diets as preventative health measures; integration with clinical practice; Emerging policy bridge between healthcare and agriculture; agroecology-linked diets show improvements in nutritional outcomes and dietary diversity | (12–15) |
| Food Security Partners Coalition (Tennessee) | 2010s–Present | Tennessee, USA |  |  |  | x |  |  | x | Advanced food security through cross-sector partnerships and local capacity building | (16) |
| Urban Agriculture Investment Programs | 2010s–Present | Various US Cities |  | x | x | x |  |  | x | Improves food access, social cohesion, biodiversity, and climate resilience in urban areas | (17–21) |
| Institutional Procurement Standards (SNAP, hospitals, schools) | Ongoing | USA (federal and local) | x | x |  |  | x |  | x | Aligns public spending with regenerative sourcing; increases market demand for RA foods | (22–25) |
| Farm Transition & Land Access Tax Incentives | Proposed | USA (potentially national) | x | x | x |  | x |  |  | Incentivizes regeneration of land and education through 4H, FFA; improves land stewardship; generational transition | (26–28) |
| CSA + Meal Kit Bundles as Prescription Programs | Proposed | USA |  |  |  |  | x | x | x | Programs pairing CSA shares with meal kits for hospitals, school meals, or “Food as Medicine” prescriptions. | (29–34) |
| 30% Local Procurement Mandates (Schools, Hospitals, Groceries, Restaurants) | Proposed | USA (potentially national) |  |  |  |  | x | x | x | Proposed mandate that 30% of institutional produce comes from locally sourced farms. | (35) |
| Zero Budget Natural Farming (ZBNF) | 2000s-Present | India | x | X | x | x | x | x | x | Agroecology/Transition Support; Scalable, low-cost regenerative approach for smallholders; improved soil health and farmer livelihoods. Utilizes FAO TAPE.* | (36,37) |
| MST Agroecology Program | 1980s-Present | Brazil | x | x | x | x | x | x | x | Land Reform/Community-Based Agroecology; Community-led adoption of agroecological practices across settlements; food sovereignty and land regeneration. Utilizes FAO TAPE.* | (15,38) |
| CGIAR Agroecology Initiative | 2021- Present | Burkina Faso, Honduras, India, Kenya, Lao PDR, Peru, Senegal, Tunisia, Zimabawe | x | x | x | x | x | x | x | International Research & Scaling; Participatory methods to integrate traditional knowledge with scientific agroecology frameworks; pilot scaling. | (39–41) |
| FAO Tool for Agroecology Performance Evaluation (TAPE) (42) | | | | | | | | | | | |

**References**

1. COMMUNICATION FROM THE COMMISSION TO THE EUROPEAN PARLIAMENT, THE COUNCIL, THE EUROPEAN ECONOMIC AND SOCIAL COMMITTEE AND THE COMMITTEE OF THE REGIONS A Farm to Fork Strategy for a fair, healthy and environmentally-friendly food system [Internet]. 2020. Available from: https://eur-lex.europa.eu/legal-content/EN/TXT/?uri=CELEX:52020DC0381

2. FAO. Global Soil Partnership 2012-2022 [Internet]. Food and Agriculture Organization of the United Nations; 2022 [cited 2025 May 27]. Available from: http://www.fao.org/documents/card/en/c/cc0921en

3. Conservation Stewardship Program | Natural Resources Conservation Service [Internet]. 2025 [cited 2025 May 27]. Available from: https://www.nrcs.usda.gov/programs-initiatives/csp-conservation-stewardship-program

4. Environmental Quality Incentives Program | Natural Resources Conservation Service [Internet]. 2025 [cited 2025 May 27]. Available from: https://www.nrcs.usda.gov/programs-initiatives/eqip-environmental-quality-incentives

5. Regenerative Organic Certified [Internet]. [cited 2025 May 27]. Farm like the World Depends on it. Available from: https://regenorganic.org/

6. CDFA - OARS - Healthy Soils Program [Internet]. [cited 2025 May 27]. Available from: https://www.cdfa.ca.gov/oars/healthysoils/

7. Farm To School [Internet]. [cited 2025 May 27]. Available from: https://www.farmtoschool.org/

8. Bronsing-Lazalde C. Center for Good Food Purchasing. [cited 2025 May 27]. The Center for Good Food Purchasing. Available from: https://goodfoodpurchasing.org/

9. Harvest New York - Cornell University - Cornell Cooperative Extension [Internet]. [cited 2025 May 27]. Available from: https://harvestny.cce.cornell.edu/

10. The Official Web Page of the United Farm Workers of America [Internet]. UFW. [cited 2025 May 29]. Available from: http://ufw.org/

11. Benavidez GA. Chronic Disease Prevalence in the US: Sociodemographic and Geographic Variations by Zip Code Tabulation Area. Prev Chronic Dis [Internet]. 2024 [cited 2025 May 29];21. Available from: https://www.cdc.gov/pcd/issues/2024/23_0267.htm

12. Food Is Medicine: A Project to Unify and Advance Collective Action | odphp.health.gov [Internet]. [cited 2025 May 29]. Available from: https://odphp.health.gov/our-work/nutrition-physical-activity/food-medicine

13. Nutrition Policy Initiative [Internet]. Food is Medicine. [cited 2025 May 29]. Available from: https://tuftsfoodismedicine.org/project/nutrition-policy-initiative/

14. Pathways to Enable Food Is Medicine Interventions [Internet]. [cited 2025 May 29]. Available from: https://odphp.health.gov/sites/default/files/2024-09/Updated-Food%20Is%20Medicine%20Pathways%20Brief%20FINAL%20508.pdf

15. James D, Blesh J, Levers C, Ramankutty N, Bicksler AJ, Mottet A, et al. The state of agroecology in Brazil: An indicator-based approach to identifying municipal “bright spots.” Elem Sci Anthr. 2023 Jun 16;11(1):00011.

16. Freedman DA, Bess KD. Food Systems Change and the Environment: Local and Global Connections. Am J Community Psychol. 2011 Jun 1;47(3):397–409.

17. Johnston J, Biro A, MacKendrick N. Lost in the Supermarket: The Corporate-Organic Foodscape and the Struggle for Food Democracy. Antipode. 2009;41(3):509–32.

18. USDA Announces Grants and Technical Assistance Funding for Urban Agriculture and Innovative Production | Home [Internet]. 2025 [cited 2025 May 29]. Available from: https://www.usda.gov/about-usda/news/press-releases/2025/01/08/usda-announces-grants-and-technical-assistance-funding-urban-agriculture-and-innovative-production

19. USDA Urban Agriculture Programs at a Glance [Internet]. [cited 2025 May 29]. Available from: https://www.fsa.usda.gov/sites/default/files/documents/USDA-Urban-Agriculture-Programs-at-a-Glance.pdf

20. Urban Agriculture and Innovative Production Grants | Home [Internet]. 2025 [cited 2025 May 29]. Available from: https://www.usda.gov/farming-and-ranching/agricultural-education-and-outreach/urban-agriculture-and-innovative-production/urban-agriculture-and-innovative-production-grants

21. Urban Agriculture and Innovative Production Fact Sheet [Internet]. [cited 2025 May 29]. Available from: https://www.usda.gov/sites/default/files/documents/farmers-urban-ag-grants-factsheet.pdf

22. Campbell C. Values-based institutional food procurement programs: A narrative review. J Agric Food Syst Community Dev. 2023 Aug 21;12(4):123–33.

23. SPUR_Overview_Good_Food_Purchasing_Toolkit.pdf [Internet]. [cited 2025 May 29]. Available from: https://www.spur.org/sites/default/files/2023-10/SPUR_Overview_Good_Food_Purchasing_Toolkit.pdf

24. Model Standards for Food Purchasing and Service.pdf [Internet]. [cited 2025 May 29]. Available from: https://www.cspinet.org/sites/default/files/2024-04/Model%20Standards%20for%20Food%20Purchasing%20and%20Service.pdf

25. Center for Science in the Public Interest [Internet]. 2024 [cited 2025 May 29]. Government food purchasing and service: Implementing best practices. Available from: https://www.cspinet.org/resource/government-food-purchasing-and-service-implementing-best-practices

26. OECD [Internet]. [cited 2025 May 29]. Food systems. Available from: https://www.oecd.org/en/topics/food-systems.html

27. USDA. Transition Incentives Program [Internet]. Available from: https://www.fsa.usda.gov/programs-and-services/conservation-programs/transition-incentives

28. Parajuli R. Present Use Value: The Basics of Agricultural and Forest Use Property Tax [Internet]. NC Extension; Available from: https://farmlaw.ces.ncsu.edu/land-use-and-zoning/present-use-value-the-basics-of-agricultural-and-forest-use-property-tax/

29. Second Harvest Heartland. FOODRx — A Food is Medicine Program [Internet]. Available from: https://www.2harvest.org/about-us/programs-services/foodrx

30. (Im)Proving the CSA Model [Internet]. Available from: https://justroots.org/impact/csaresearch/

31. Berkowitz SA, O’Neill J, Sayer E, Shahid NN, Petrie M, Schouboe S, et al. Health Center–Based Community-Supported Agriculture: An RCT. Am J Prev Med. 2019 Dec 1;57(6):S55–64.

32. Food is Medicine Coalition & Center for Health Law and Policy Innovation. Food is Medicine: A State Medicaid Policy Toolkit [Internet]. 2024. Available from: https://fimcoalition.org/wp-content/uploads/2024/07/Food-is-Medicine-A-State-Medicaid-Policy-Toolkit_Final-July-2024-1.pdf

33. Wholesome Wave. Produce Prescription Program [Internet]. Available from: https://www.wholesomewave.org/what-we-do-1

34. NourishedRx. Food is health, food is care [Internet]. Available from: https://www.nourishedrx.com/

35. New York State Department of Agriculture and Markets. 30% New York State Initiative [Internet]. 2021. Available from: https://agriculture.ny.gov/30-percent-initiative

36. Khadse A, Rosset PM, Morales H, Ferguson BG. Taking agroecology to scale: the Zero Budget Natural Farming peasant movement in Karnataka, India. J Peasant Stud. 2018 Jan 2;45(1):192–219.

37. Krishnan S, Gupta S, Malaiappan S, Singh S, Kumar G, Alvi M, et al. Performance Assessment of Agroecology in India. CGIAR; 2024 Dec.

38. Pettan-Brewer C, Martins AF, Abreu DPB de, Brandão APD, Barbosa DS, Figueroa DP, et al. From the Approach to the Concept: One Health in Latin America-Experiences and Perspectives in Brazil, Chile, and Colombia. Front Public Health [Internet]. 2021;Volume 9-2021. Available from: https://www.frontiersin.org/journals/public-health/articles/10.3389/fpubh.2021.687110

39. Quintero M, Wiederkehr Guerra GP, Staiger Rivas S, Dickens C. CGIAR Initiative on Agroecology. 2023 May [cited 2025 May 29]; Available from: https://hdl.handle.net/10568/130662

40. FAO. Evaluation of FAO’s contributions to Sustainable Development Goal 2 - Agroecology [Internet]. Rome; 2021. Available from: https://openknowledge.fao.org/server/api/core/bitstreams/48b7f759-cc51-4fd2-b00b-f5e73f3dde5d/content

41. Voss RC, Freed S, Falk T, Rietveld A, Rahma A, Alary V, et al. Agency and behavior change in agri-food systems transformation: Lessons from the CGIAR Agroecology Initiative [Internet]. 2024 p. 44. Available from: https://hdl.handle.net/10568/141843

42. Tool for Agroecology Performance Evaluation (TAPE) - Test version [Internet]. [cited 2025 May 30]. Available from: https://openknowledge.fao.org/items/8511c796-c7d1-4a04-895d-a28115731ce0

**Supplementary Fig.1. The three-phase flow diagram outlining literature and report assessment for review inclusion**

Records identified from:

Websites (n = 40)

Organizations (n = 30)

Records removed *before screening*:

Duplicate records removed (n = 15)

Records removed as outside scope of review (n = 5)

Records identified from*:

Databases (n = 3)

**Identification**

Records screened

(n = 349)

Records excluded**

(n = 199)

Reports not retrieved

(n = 8)

Reports sought for retrieval

(n = 70)

Reports sought for retrieval

(n = 150)

Reports not retrieved

(n = 10)

**Screening**

Reports excluded: (n = 0)

Reports assessed for eligibility

(n = 62)

Reports assessed for eligibility

(n = 140)

Reports excluded:

Outside scope (n = 20)

Incomplete data (n = 11)

Studies/Reviews included in review

(n = 109)

Reports of included studies

(n = 62)

**Included**

Source: Page MJ, et al. BMJ 2021;372:n71. doi: 10.1136/bmj.n71.This work is licensed under CC BY 4.0. To view a copy of this license, visit <https://creativecommons.org/licenses/by/4.0/>
